# KLS-3021, a multifunctional oncolytic virus, demonstrates potent early-intervention efficacy in preclinical models of prostate-confined and locally advanced prostate cancer

**DOI:** 10.3389/fonc.2026.1765625

**Published:** 2026-02-13

**Authors:** Jinwon Seo, Kiwon Park, Sungmin Lee, Hyesoo Kang, Joonsung Kim, Eunjin Lee, Jaeil Shin, Soon-Oh Hong, Sujeong Kim, Sun Jin Kim

**Affiliations:** Institute of BioInnovation Research, Kolon Life Science, Inc., Seoul, Republic of Korea

**Keywords:** oncolytic virotherapy, immunotherapy, prostate cancer, orthotopic tumor mice model, PH-20, IL-12, sPD1-Fc

## Abstract

**Introduction:**

With aging male populations, the incidence of prostate cancer is increasing globally. Patients with low- to intermediate-risk disease (Gleason score ≤7) are often managed with close surveillance rather than immediate curative intervention; however, progression in a subset of these individuals narrows the window for effective treatment. Minimally invasive, image-guided local therapy enabling early intervention could prevent disease progression and improve progression-free outcomes. To address this unmet need, we evaluated KLS-3021, a novel oncolytic vaccinia virus encoding PH-20, IL-12, and soluble PD1-Fc.

**Methods:**

KLS-3021 was assessed in two orthotopic prostate cancer models: a prostate-confined model with bioluminescence-positive, non-gross tumors, and a visible/palpable locally invasive model with regional lymphatic spread. Mice were randomized to receive vehicle, docetaxel, or KLS-3021, administered on day 9 (prostate-confined) or day 29 (visible/locally invasive) after implantation of luciferase-labeled PC-3 cells. Tumor growth was tracked longitudinally by bioluminescence imaging, and histopathological analyses were conducted at predefined time points.

**Results:**

A single intratumoral injection of KLS-3021 induced profound and durable tumor therapeutic or suppressive efficacy superior to that of docetaxel in prostate-confined or locally invasive diseases. Mechanistic analyses revealed extracellular matrix degradation, enhanced viral spread, increased immune cell infiltration, M1 macrophage polarization, and features of immunogenic cell death in KLS-3021-treated tumors.

**Discussion:**

These results collectively demonstrate the translational potential of KLS-3021 as a minimally invasive or neoadjuvant therapeutic strategy for prostate cancer, especially in patients for whom early, localized intervention is medically feasible.

## Introduction

Prostate cancer is one of the most commonly diagnosed malignancies in men, with incidence and prevalence rising globally due to aging populations and environmental factors such as diet and lifestyle (1). A distinctive feature of its management is that many patients, particularly those with low-risk diseases stratified by Gleason score and genomic risk classifiers, are managed through active surveillance without immediate medical intervention (2, 3). Although most of these tumors progress slowly and do not affect the overall lifespan, disease progression can occur unpredictably in a subset of patients. In some cases, localized tumors advance to life-threatening systemic diseases, presenting a particularly difficult challenge for patients who were initially managed conservatively, especially given the lack of reliable biomarkers to predict progression (4).

Although radical prostatectomy, including robotic-assisted techniques, and radiation therapy remain the cornerstones of curative treatment for localized prostate cancer, these modalities are inherently invasive and frequently associated with substantial morbidity (5). Surgical resection poses heightened perioperative risks in older patients and those with frailty or comorbidities, and radiation therapy can result in cumulative toxicities that significantly impair quality of life, including urinary incontinence, erectile dysfunction, and gastrointestinal injury (6). Despite advances in precision planning and delivery, these side effects often outweigh the potential benefits in older or medically ineligible patients. Minimally invasive focal approaches, such as high-intensity focused ultrasound (HIFU) and cryoablation, have emerged as alternatives but remain limited by challenges in accurately delineating tumor margins and achieving complete ablation, resulting in variable recurrence rates (7, 8). Consequently, a pressing unmet need for novel, localized, and organ-preserving therapeutic strategies remains, particularly for patients with prostate-confined or locally invasive mid- to low-grade cancer that transcends conventional modalities such as surgery, radiation, or isotope therapy. Such approaches should provide effective tumor control with minimal systemic burden, especially in aging or medically ineligible populations.

Oncolytic viruses (OVs) represent a promising therapeutic platform that integrates direct tumor lysis with potent immune reprogramming (9). In prostate cancer, where immune checkpoint blockade (ICB) has shown limited efficacy (10), OVs offer a rational strategy to remodel local immunity while simultaneously targeting tumor cells. We previously developed KLS-3020, a recombinant oncolytic vaccinia virus that demonstrated potent tumor-suppressive effects and immune activation in animal models (11). By removing reporter genes, we subsequently generated KLS-3021, a clinically optimized version designed for translational applications. KLS-3021 encodes human PH-20 hyaluronidase to degrade the dense tumor extracellular matrix (ECM) (12), IL-12 to promote innate and adaptive immune activation (13), and soluble PD1-Fc (sPD1-Fc) to locally inhibit immune checkpoint signaling without inducing systemic toxicity (14).

We evaluated the therapeutic potential of KLS-3021 in orthotopic prostate cancer models representing two clinically relevant stages: signal-positive tumors corresponding to prostate-confined disease, detectable only by an *in vivo* imaging system (IVIS), and visible tumors mimicking locally advanced disease with regional lymphatic involvement. We further investigated whether KLS-3021 promoted viral spread, enhanced immune cell infiltration, and induced immunogenic cell death (ICD). These findings support the translational potential of KLS-3021 as a minimally invasive or neoadjuvant therapeutic candidate with the potential to improve outcomes for patients with prostate-confined or locally advanced prostate cancer, particularly those who are older or medically unfit.

## Methods

### Cell culture

Human prostate cancer PC-3 cells (Cat. No. KCLB 21435, RRID. CVCL_0035) were purchased from the Korean Cell Line Bank (KCLB). Luciferase-labeled PC-3 cells were generated for bioluminescence-based tumor monitoring. Cells were transduced with a lentiviral vector encoding firefly luciferase under the CMV promoter (LV-CMV–Firefly luciferase, Kerafast, FCT006), and stable clones were established through puromycin selection. The resulting luciferase-labeled PC-3 cells were maintained in RPMI 1640 medium (HyClone) supplemented with 10% fetal bovine serum (FBS, HyClone) and 1% penicillin–streptomycin (Gibco) and incubated at 37 °C in a mixture atmosphere of 5% CO_2_ and 95% air. The cell line was authenticated *via* short tandem repeat profiling (data not shown).

### Plasmid construction

To engineer recombinant vaccinia viruses, shuttle plasmids targeting the C11R, K3L, and J2R loci were constructed. Briefly, cDNAs encoding PH-20 and sPD1-Fc were inserted into the J2R shuttle plasmid under the control of the Hyb and 7.5 promoters, respectively, between approximately 1 kb homologous arms flanking the J2R gene. Similarly, IL-12 cDNA was placed downstream of the I1L–B19R early/late promoter within the K3L shuttle plasmid, positioned between ~1 kb left and right flanking regions of the K3L gene. All plasmid constructs were confirmed by restriction enzyme mapping and Sanger sequencing.

### Generation of recombinant vaccinia virus

KLS-3021 was generated by homologous recombination (HR). Briefly, HeLa cells were infected with previously generated KLS-3010 (15) at a multiplicity of infection (MOI) of 0.05, and transfected with the C11R-targeting shuttle plasmid 15 min post-infection. After 4 h, the medium was replaced with fresh Dulbecco’s modified Eagle’s medium containing 5% FBS, and cells were incubated for 48 h at 37 °C in a humidified 5% CO_2_ atmosphere. The cells were then collected, subjected to three freeze–thaw cycles, and the supernatant containing recombinant virions was harvested. Recombinant viral clones were isolated *via* repeated rounds of plaque purification and confirmed by polymerase chain reaction using locus-specific primers. Viral genomic DNA was extracted using either the Maxwell^®^ Viral Total Nucleic Acid Purification Kit (Promega) or the NucleoSpin^®^ RNA/DNA Virus Kit (MN). The final recombinant virus, designated KLS-3021, was constructed by sequentially deleting the C11R and J2R loci, followed by replacement of the K3L locus using the double-deleted virus as the template.

### Orthotopic prostate cancer model

All animal experiments were approved by the Kolon Pharma Institutional Animal Care and Use Committee (approval no. KPLARC-IACUC-25-001). Six-week-old male BALB/c nude mice (JA BIO, Inc.) were used.

The luciferase-labeled PC-3 cells were harvested and resuspended at a final concentration of 1.0 × 10^5^ cells in 50 μL Ca^2+^/Mg^2+^ free Hank’s balanced salt solution (HBSS, Gibco). Mice were anesthetized by intramuscular injection of a Zoletil (Virbac)/Rompun (Bayer) mixture prepared at a 3:1 ratio at a dose of 1 mL/kg per mouse. For the orthotopic prostate cancer model, a lower midline abdominal incision was made to expose the prostate, and luciferase-labeled PC-3 cells were directly injected into the right dorsal lobe using a 30-gauge needle. The prostate was repositioned, and the incision was closed using wound clips (Clay Adams). Tumor growth was monitored once a week using the IVIS^®^ Lumina LT series III *In Vivo* Imaging System (PerkinElmer).

Two distinct orthotopic prostate cancer models were established: a prostate-confined and a locally invasive model. For the prostate-confined model (bioluminescence-positive, non-palpable tumors), mice were selected based on the absence of grossly visible tumors but the presence of IVIS-detectable bioluminescence following intraperitoneal D-luciferin administration, simulating surveillance-eligible, organ-confined disease (Gleason score ≤ 7). For the locally invasive model (gross tumors with regional lymphatic spread), mice were selected based on the presence of palpable prostate tumors accompanied by gross evidence of local invasion and regional lymph node involvement on bioluminescence imaging.

### Treatment protocol

In both models, mice were randomly assigned to receive one of three treatments: formulation buffer, KLS-3021, or docetaxel (DITAXEL 1, Boryong). Formulation buffer and KLS-3021 (1×10^7^ TCID_50_/head, 20 μL) were administered *via* intratumoral injection, whereas docetaxel was administered intraperitoneally at 10 mg/kg once weekly for seven and four cycles in the prostate-confined and locally invasive models, respectively. Treatments were initiated on day 9 (prostate-confined model) or 29 (locally invasive model) after tumor cell implantation. For histological analysis, a subset of mice was euthanized on day 3 post-treatment, and the remaining animals were sacrificed at the experimental endpoint. Mice designated for tissue sampling were anesthetized by intramuscular injection of a Zoletil/Rompun mixture as described above, followed by organ collection. These animals were subsequently euthanized by exsanguination through transection of both the abdominal aorta and vena cava. Mice not subjected to tissue sampling were anesthetized using the same procedure and euthanized by carbon dioxide (CO_2_) inhalation, with CO_2_ introduced into the chamber at a controlled displacement rate of 20–30% of the chamber volume per minute until death was confirmed, in accordance with generally accepted guidelines.

### *In vivo* bioluminescence imaging

Tumor growth was monitored weekly *via* bioluminescence imaging (IVIS Lumina III; PerkinElmer). For imaging, mice were intraperitoneally administered D-luciferin (150 mg/kg, PerkinElmer), and a full-body IVIS scan was performed approximately 10 min post-administration. Mice were anesthetized by inhalation of isoflurane (3%) delivered in oxygen at a flow rate of 2.5 L/min. Imaging parameters were standardized across all sessions, and photon flux was quantified using Living Image software (PerkinElmer).

### Histological and immunohistochemical analysis

Prostate tissues and lumbar lymph nodes were harvested, fixed in 10% neutral-buffered formalin, and embedded in paraffin. For histological examination, 4 μm sections were prepared and stained with hematoxylin and eosin (H&E, BBC Biochemical) for both tissue types, whereas Alcian blue staining (Abcam) was performed on prostate tissues according to the manufacturer’s protocols.

For immunohistochemistry, sections were treated with peroxidase blocking solution (Dako) to quench endogenous peroxidase activity and then incubated with 4% fish gelatin (Aurion) to block nonspecific binding. Subsequently, sections were incubated with vaccinia virus (Abcam, ab35219) or F4/80 (Abcam, ab111101, AB_10859466) antibody, followed by horseradish peroxidase-labeled polymer (Dako). Signals were developed using diaminobenzidine (Dako), counterstained with Gill-2 hematoxylin (StatLab), and mounted with Optic-Mount xylene-based medium (Statab). Images were acquired using a Leica DM2500 microscope equipped with a digital camera (Leica Microsystems).

### Immunofluorescence staining and TUNEL assay

Harvested tissues were fixed in 4% paraformaldehyde, embedded in optimal cutting temperature (Sakura) compound, and sectioned at 7 μm. Sections were blocked with 4% fish gelatin and incubated with primary antibodies, followed by fluorophore-conjugated secondary antibodies.

For single immunofluorescence, primary antibodies included CD86 (Invitrogen, Cat. No. 14-0862-82, RRID. AB_467368), CD206 (Abcam, Cat. No. ab64693, RRID. AB_1523910), TNF-α (Abcam, Cat. No. ab307164, RRID. AB_2941996), HMGB1 (Abcam, Cat. No. ab18256, RRID. AB_444360), and calreticulin (Abcam, Cat. No. ab92516, RRID. AB_10562796). Alexa Fluor 488–conjugated secondary antibodies (Invitrogen, Cat. No. A-11006 for CD86; Abcam, Cat. No. ab150077 for all others) were used for detection.

For double immunofluorescence, tissues were stained with Ki-67 (Abcam, Cat. No. ab15580, RRID. AB_443209) and CD31 (BD Pharmingen, Cat. No. 553370, RRID. AB_394816) primary antibodies, followed by Alexa Fluor 488-conjugated (Invitrogen, Cat. No. A-11034) and Alexa Fluor 594-conjugated (Invitrogen, Cat. No. A-11037) secondary antibodies, respectively. The TUNEL assay was performed using the DeadEnd Fluorometric TUNEL System (Promega, Cat. No. G3250) according to the manufacturer’s instructions, followed by co-immunostaining with CD31 (BD Pharmingen) and Alexa Fluor 594 (Invitrogen). Fluorescence images were captured using a ZEISS Axio Scan Z1 slide scanner under identical exposure settings.

### Statistical analysis

Quantitative data are expressed as the mean ± standard error of the mean (SEM). Statistical analyses were performed using GraphPad Prism version 10 (GraphPad Software, USA). For multiple group comparisons, data were first assessed for normality using the Shapiro–Wilk test. Normally and non-normally distributed data were analyzed using one-way analysis of variance (ANOVA), followed by Dunn’s multiple comparison test, and the Kruskal–Wallis test, respectively. The specific statistical tests used for each dataset are described in the corresponding figure legends.

## Results

### KLS-3021 induces durable tumor growth inhibition in a signal-positive orthotopic prostate cancer model representing prostate-confined disease

**Figure 1:**
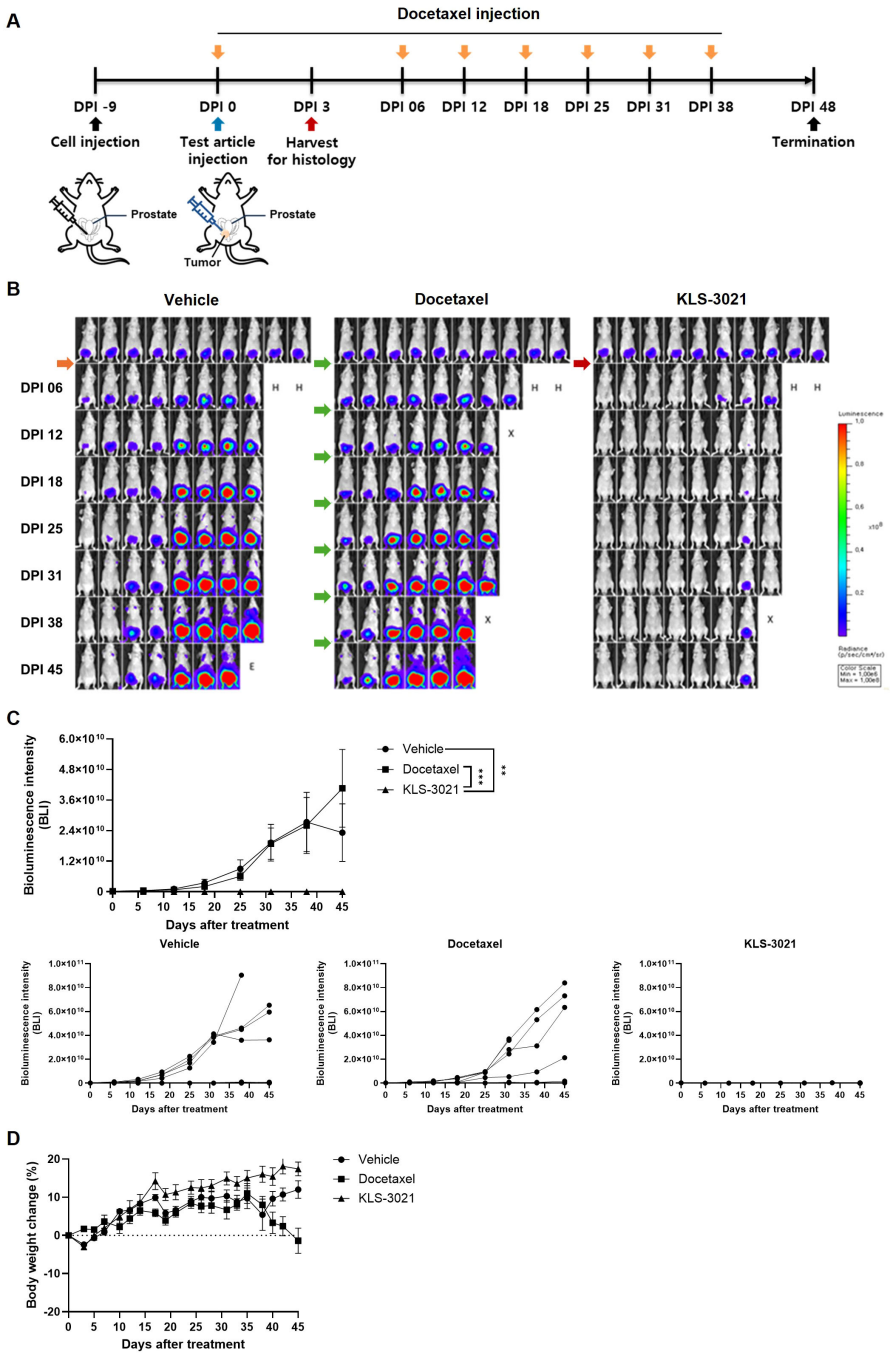
Single intratumoral KLS-3021 administration results in significant tumor growth inhibition in a prostate-confined orthotopic cancer model. **(A)** Schematic diagram illustrating the experimental design. **(B)** Representative bioluminescence IVIS images of mice from each treatment group at the indicated time points. Symbols indicate the experimental status of each mouse: H, animals harvested for histological analysis; X, animals found dead; E, animals euthanized for humane endpoints. **(C)** Quantification of bioluminescence signal intensity over time. Top, mean signal intensity per group (n = 8 per group; mean ± SEM). Bottom, individual mouse signal intensities displayed. **(D)** Body weight changes over time in each treatment group (mean ± SEM, n = 8 per group). Statistical significance was determined using one-way ANOVA with post-hoc tests. ** indicates *p* < 0.01 and *** indicates *p* < 0.001.

To establish a preclinical model simulating early-stage, prostate-confined cancer representative of patients with Gleason score ≤7 under active surveillance, luciferase-labeled PC-3 cells were injected into the right dorsal lobe of the prostate in BALB/c nude mice (**Figure 1A**). Nine days post-injection, mice exhibiting bioluminescent signals without palpable tumors were selected, reflecting a clinical scenario in which tumor burden is detectable by imaging but not by physical examination.

Selected mice received a single intraprostatic injection of vehicle or KLS-3021 (1x10^7^ TCID_50_/head). As a comparator, the standard-of-care agent docetaxel was administered intraperitoneally once weekly for seven cycles. For histological evaluation of early treatment effects, a subset of animals was sacrificed on day 3 post-treatment, and the remaining mice were monitored longitudinally until sacrifice on day 45.

Whole-body bioluminescence imaging using IVIS revealed robust tumor control in the KLS-3021 group (**Figure 1B**). Unlike the persistent or escalating bioluminescence observed in the vehicle and docetaxel groups, KLS-3021-treated mice exhibited rapid, profound, and durable tumor signal suppression, indicating robust tumor growth inhibition following a single intraprostatic injection, and highlighting the potent and lasting anti-tumor activity of KLS-3021. Quantitative analysis further confirmed that bioluminescence intensity was significantly lower in KLS-3021–treated mice at all measured time points than in both control groups (**Figure 1C**), with consistent responses observed across individual animals. Body weight remained stable throughout the study in the KLS-3021 treatment group (**Figure 1D**).

Collectively, these results indicated that a single intraprostatic injection of KLS-3021 achieved sustained tumor growth inhibition in a model representative of signal-positive, prostate-confined disease, supporting its potential as a localized therapeutic strategy for patients under active surveillance.

### KLS-3021 suppresses tumor growth and lymphatic metastasis in a prostate-confined orthotopic cancer model

**Figure 2:**
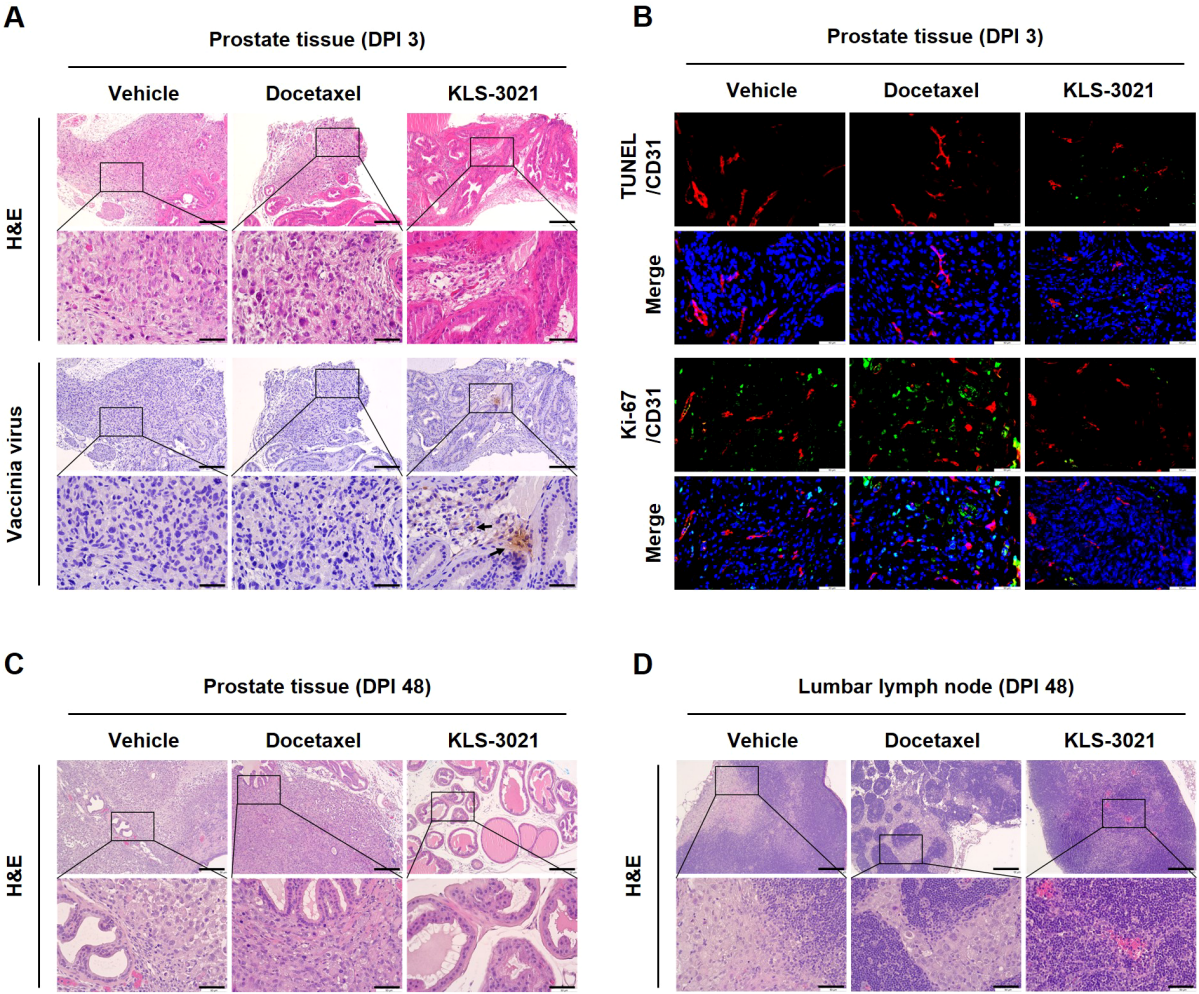
Histological analysis showed suppression of tumor growth and nodal metastasis following KLS-3021 treatment in a prostate-confined orthotopic cancer model. **(A)** H&E and vaccinia virus immunohistochemistry staining of prostate tissue sections collected on day 3 post-treatment. **(B)** TUNEL/CD31 and Ki-67/CD31 immunofluorescence staining of prostate tissues on day 3 post-treatment. H&E-stained prostate tissue sections **(C)** and lumbar lymph nodes **(D)** collected on day 48 post-treatment. Scale bars, 200 μm. Enlarged scale bars, 50 μm. Representative images from two mice per group are shown.

Given the robust tumor growth inhibition observed *via* bioluminescence imaging, we performed histological analyses to evaluate the structural effects of KLS-3021 on both primary and nodal sites. H&E staining of prostate tissues on day 3 post-treatment revealed distinct differences among the treatment groups. In both vehicle- and docetaxel-treated mice, tumors retained dense cellularity, indicating sustained tumor growth. Conversely, KLS-3021-treated prostates exhibited a near-normal architecture with markedly reduced tumor cell density (**Figure 2A**). Immunohistochemical staining for vaccinia virus demonstrated localized viral presence in the residual tumor regions of KLS-3021-treated prostates, suggesting effective viral delivery and engagement within the tumor microenvironment (TME).

To elucidate the mechanism underlying tumor regression, we performed immunofluorescence staining for TUNEL, Ki-67, and CD31 to assess tumor cell death–associated DNA fragmentation, cellular proliferation, and vascular density. In the KLS-3021-treated group, TUNEL-positive apoptotic cells were markedly increased compared with those in the vehicle- and docetaxel-treated groups. Conversely, Ki-67-positive proliferating tumor cells and CD31-positive vascular structures were notably decreased (**Figure 2B**). These findings indicate that KLS-3021 suppresses tumor growth by promoting tumor cell death and inhibiting tumor cell proliferation. By day 48, this early cellular disruption culminated in near-complete tumor ablation, with histopathological analysis of the prostate gland revealing tissue architecture consistent with normal histology (**Figure 2C**). Conversely, the vehicle- and docetaxel-treated groups retained extensive regions of viable tumors. Notably, in the docetaxel group, residual tumor areas were indicative of suboptimal or delayed therapeutic efficacy. Similar histological patterns were observed in the tumor-draining lumbar lymph nodes (**Figure 2D**). Although metastatic tumor foci were readily detectable in the control groups, no evidence of tumor infiltration was found in the lymph nodes of KLS-3021-treated mice, indicating effective suppression of regional metastatic dissemination.

These data suggested that KLS-3021 exerts potent and multifaceted anti-tumor effects by initiating localized viral activity, triggering tumor cell death, and suppressing tumor cell proliferation. Notably, this single-dose intervention was sufficient to eliminate the primary tumor and prevent lymphatic dissemination, underscoring its potential as a durable and localized treatment modality for prostate cancer.

### KLS-3021 exerts potent therapeutic efficacy and achieves sustained near-complete regression in a locally invasive orthotopic prostate cancer model

**Figure 3:**
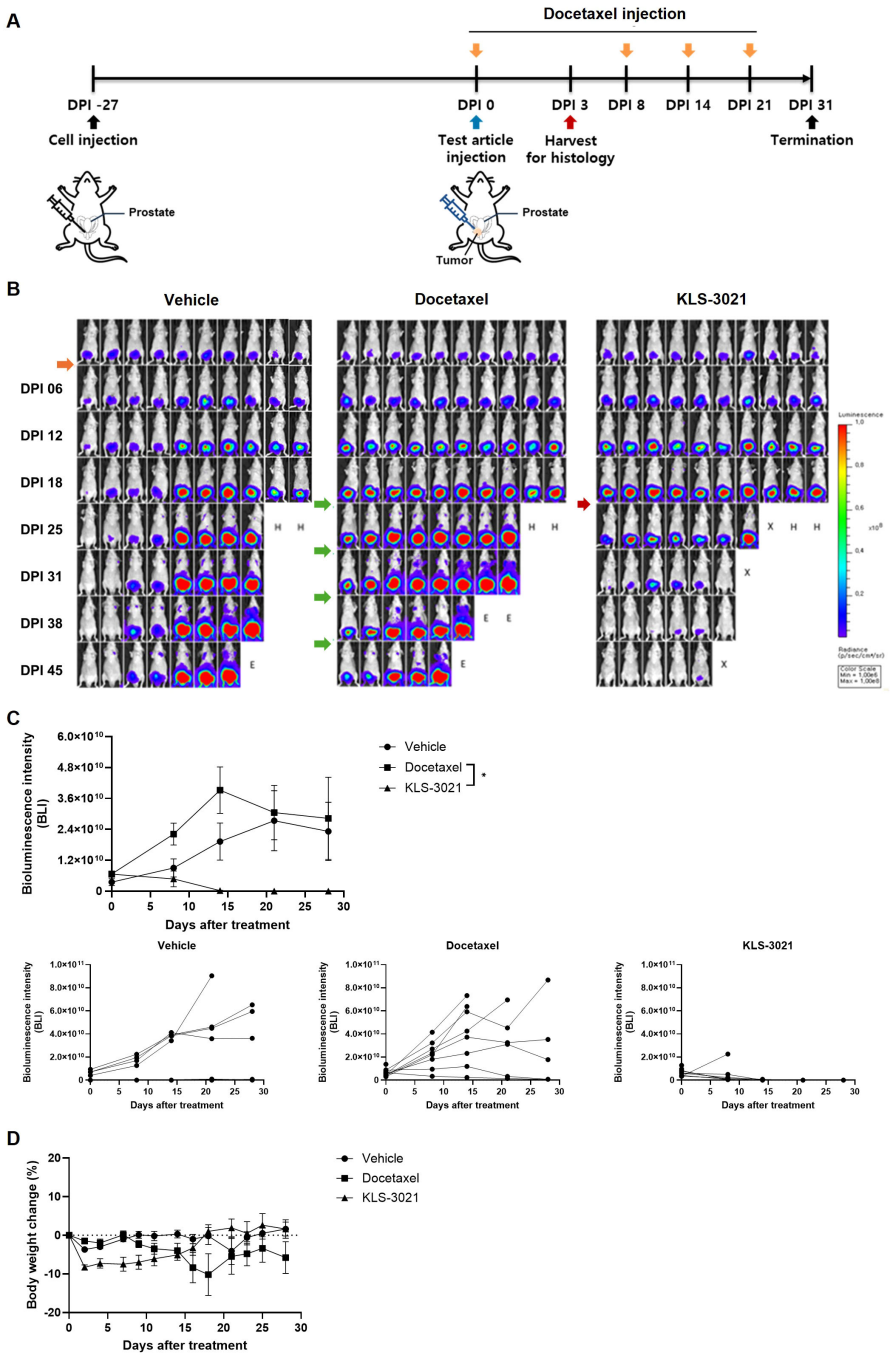
Single intratumoral injection of KLS-3021 elicits potent anti-tumor activity and sustained tumor regression in a locally invasive orthotopic prostate cancer model. **(A)** Schematic diagram illustrating the experimental design. **(B)** Representative bioluminescence IVIS images of mice from each treatment group at the indicated time points. Symbols indicate the experimental status of each mouse: H, animals harvested for histological analysis; X, animals found dead; E, animals euthanized for humane endpoints. **(C)** Quantification of bioluminescence signal intensity over time. Top, mean signal intensity per group (n = 8 per group; mean ± SEM). Bottom, individual mouse signal intensities displayed. **(D)** Body weight changes over time in each treatment group. Statistical significance was determined using one-way ANOVA with post-hoc tests. *p < 0.05.

Building on the strong efficacy observed in signal-positive, low-burden tumors, we evaluated KLS-3021 in a locally invasive orthotopic model characterized by palpable tumor mass and regional lymphatic spread (**Figure 3A**). Orthotopic prostate tumor-bearing mice were randomized to receive a single intratumoral injection of vehicle or KLS-3021 (1×10^7^ TCID_50_/head) or four weekly cycles of intraperitoneal docetaxel injection (10 mg/kg).

In both vehicle- and docetaxel-treated mice, bioluminescence imaging revealed progressive tumor growth over time, with increasing signal intensity and evidence of metastasis to organs beyond the prostate (**Figure 3B**). In contrast, a single intratumoral injection of KLS-3021 induced a pronounced and sustained decrease in the tumor signal, resulting in near-complete regression during the study period (**Figure 3B**). Quantitative signal analysis confirmed a significant reduction in tumor burden in the KLS-3021 group compared with that in both control groups at all measured time points (**Figure 3C**). Furthermore, individual mouse tracking revealed consistent tumor regression, with most animals displaying near-complete or complete signal loss, underscoring the durable therapeutic effects of KLS-3021.

During the study period, three animals in the KLS-3021-treated group died on days 2, 9, and 23 following intratumoral administration, whereas no deaths were observed in the vehicle- or docetaxel-treated groups (**Figure 3B**). These deaths occurred at variable time points and were not preceded by overt prodromal signs, such as rapid body weight loss, marked behavioral changes, or other clinical symptoms suggestive of systemic toxicity. Post-mortem gross examinations were performed in all three animals. In two animals (DPI 2 and DPI 23), marked enlargement of the urinary bladder with urinary retention and seminal vesicles was observed. In one animal (DPI 9), a large volume of fluid was noted and considered consistent with urinary leakage secondary to bladder rupture. Based on these findings, the observed mortality was considered most consistent with bladder neck obstruction and subsequent uremia or peritonitis due to leaked urine rather than test article–related toxicity. Bladder outlet obstruction is often observed in orthotopic primary prostate cancer models (16). All treatment groups maintained stable body weights throughout the experiment (**Figure 3D**); however, KLS-3021-treated mice exhibited a trend toward body weight gain compared with those receiving docetaxel. These findings indicate that the potent anti-tumor effects of KLS-3021 are not accompanied by overt systemic toxicity and may offer a more favorable tolerability profile than conventional chemotherapy.

Collectively, these results highlight the robust efficacy of KLS-3021 in the treatment of advanced visible prostate cancer. The ability to achieve such therapeutic benefits after a single localized injection reinforces the translational value of KLS-3021 in patients with locally advanced disease.

### KLS-3021 remodels the TME by disrupting the ECM structure and reprogramming the immune landscape in a locally invasive orthotopic prostate cancer model

To explore whether KLS-3021 modulates the TME beyond its direct oncolytic effects, prostate tumors were analyzed on day 3 post-treatment in the locally invasive orthotopic prostate cancer model.

**Figure 4:**
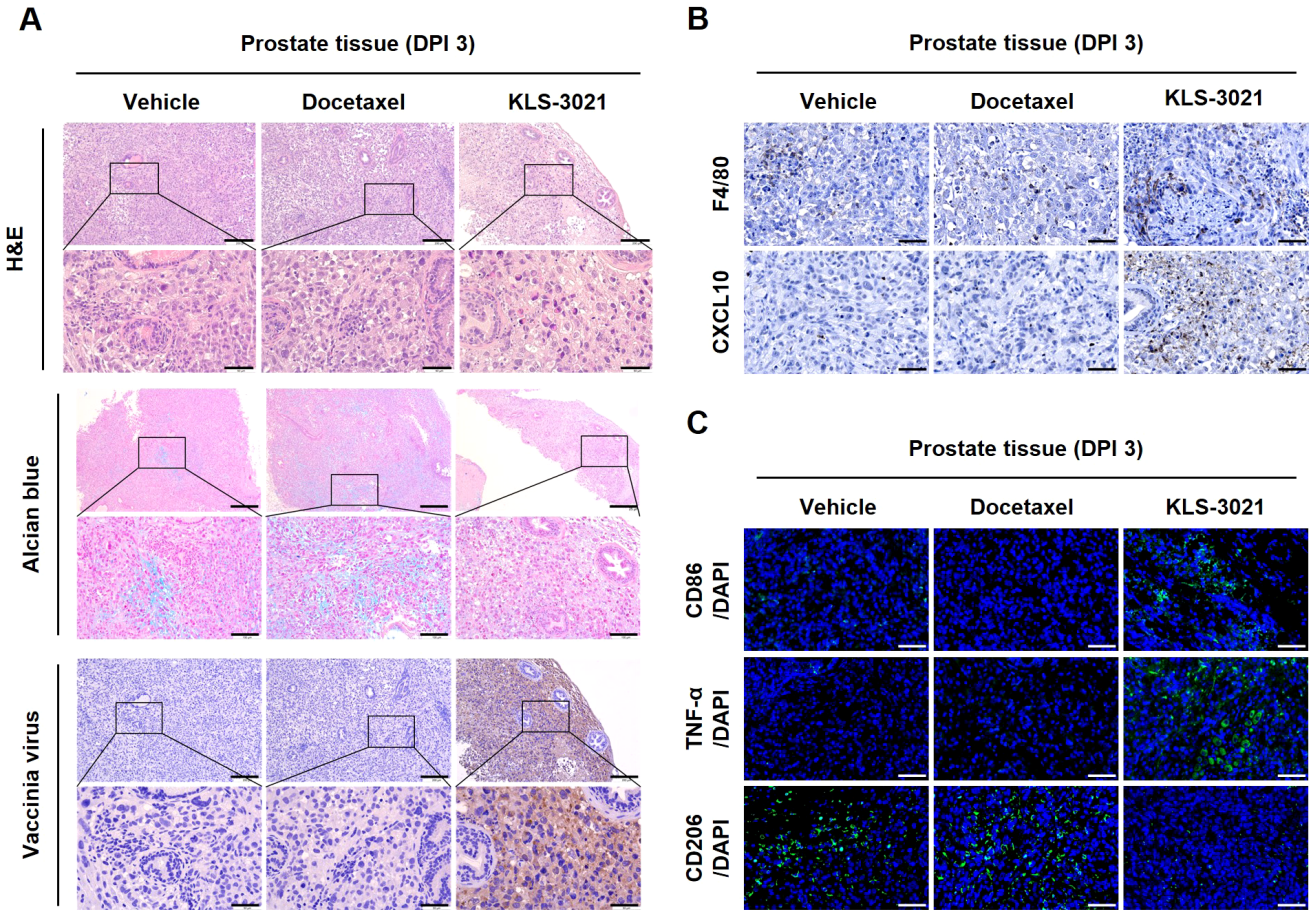
KLS-3021 treatment disrupts the tumor matrix, enhances viral dissemination, and promotes polarization toward an M1 macrophage phenotype in the locally invasive orthotopic prostate cancer model. **(A)** H&E, Alcian blue staining, vaccinia virus immunohistochemistry of prostate tissues on day 3 post-treatment. **(B)** Immunohistochemical staining of F4/80 and CXCL10 in prostate tissues. **(C)** Immunofluorescence staining of CD86, TNF-α, and CD206 in prostate tissues. Scale bars, 200 μm. Enlarged scale bars, 50 μm. Representative images from two mice per group are shown.

H&E staining revealed extensive tumor cell death and marked loss of cellular density in KLS-3021-treated tumors, whereas vehicle- and docetaxel-treated tumors retained viable tumor cells with preserved stromal structures (**Figure 4A**). Given that KLS-3021 encodes human PH-20 hyaluronidase, which degrades hyaluronic acid (HA), a major ECM component and physical barrier in solid tumors, ECM integrity was examined using Alcian blue staining. KLS-3021-treated tumors exhibited a pronounced reduction in Alcian blue-positive regions, indicating PH-20–mediated degradation of HA-rich ECM, whereas vehicle- and docetaxel-treated tumors retained dense Alcian blue-positive stroma (**Figure 4A**). Consistent with ECM degradation, vaccinia virus immunohistochemistry revealed widespread viral dissemination throughout the tumor parenchyma in the KLS-3021 group, suggesting that matrix disruption facilitated efficient intratumoral viral spread following a single administration (**Figure 4A**).

Parallel to these structural changes, KLS-3021 treatment markedly reshaped the immune landscape within the TME. Immunohistochemical analysis showed a pronounced increase in F4/80^+^ macrophage infiltration accompanied by elevated CXCL10 expression in KLS-3021-treated tumors, suggesting that CXCL10 produced by activated macrophages may facilitate the recruitment of circulating T cells into the TME (**Figure 4B**). Conversely, only a limited number of F4/80^+^ cells were detected in vehicle- and docetaxel-treated tumors. To further characterize macrophage polarization and activation status, immunofluorescence analysis for CD86, TNF-α, and CD206 was performed. KLS-3021 treatment induced a pronounced shift toward an M1 macrophage phenotype, characterized by increased CD86^+^ and TNF-α^+^ populations, accompanied by a significant reduction in CD206^+^ M2 macrophages associated with tumor progression and immunosuppression (**Figure 4C**). Conversely, vehicle- and docetaxel-treated tumors exhibited macrophage infiltration that was predominantly CD206^+^, reflecting an M2-skewed, immunosuppressive phenotype.

Collectively, these findings demonstrate that a single intratumoral injection of KLS-3021 dismantles the structural ECM barrier *via* PH-20-mediated HA degradation and facilitates widespread viral dissemination, while concurrently enhancing immune cell infiltration and reprogramming tumor-associated macrophages toward an activated, proinflammatory, M1-dominant phenotype. This combined remodeling of the structural and immune components of the TME establishes a proinflammatory anti-tumor niche that reinforces the durable therapeutic efficacy of KLS-3021.

### KLS-3021 triggers immunogenic tumor cell death with suppressed proliferation in a locally invasive orthotopic prostate cancer model

Following extensive KLS-3021-induced immune and structural remodeling in the locally invasive cancer model, we investigated whether these changes were accompanied by tumor cell death associated with ICD-related features. ICD is characterized by the release or exposure of damage-associated molecular patterns (DAMPs) that can promote anti-tumor immune responses.

**Figure 5:**
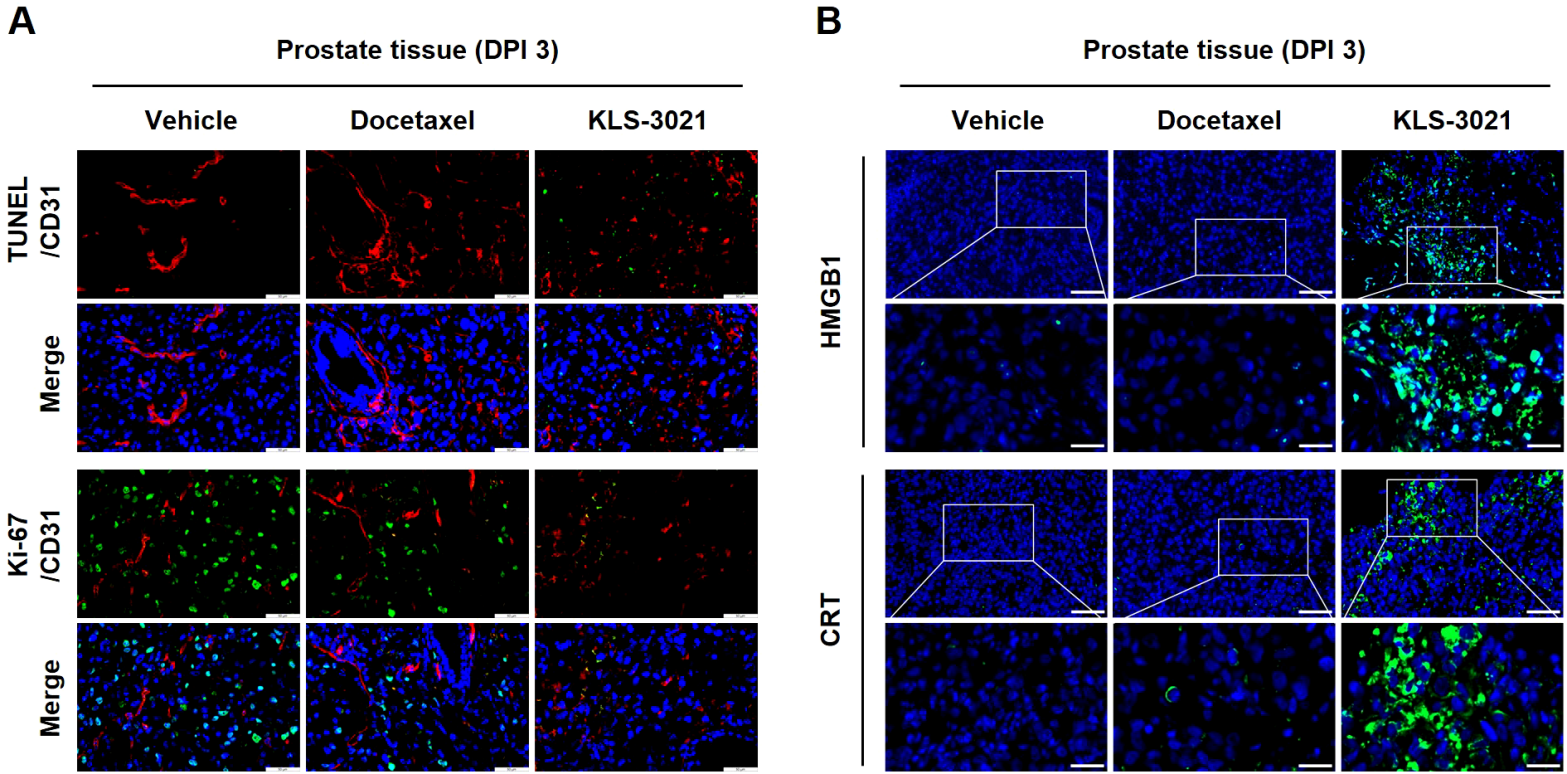
KLS-3021 treatment induces tumor cell death associated with immunogenic features, suppresses tumor cell proliferation, and promotes DAMP exposure in a locally invasive orthotopic prostate cancer model. **(A)** TUNEL/CD31 and Ki-67/CD31 immunofluorescence staining of prostate tissues collected on day 3 post-treatment. **(B)** Immunofluorescence staining of HMGB1 and CRT with DAPI. Scale bars, 50 μm. Enlarged scale bars, 20 μm. Representative images from two mice per group are shown.

On day 3 post-treatment, TUNEL staining showed a pronounced increase in TUNEL-positive signals in KLS-3021-treated tumors compared with both vehicle- and docetaxel-treated controls, indicating effective tumor cell death accompanied by DNA fragmentation (**Figure 5A**). Co-staining with CD31 showed that TUNEL-positive regions exhibited reduced vascular structures, suggesting loss of tumor vasculature in areas undergoing cell death. In parallel, Ki-67 immunofluorescence showed a marked reduction in proliferating tumor cells following KLS-3021 treatment, whereas strong Ki-67 positivity persisted in the control groups (**Figure 5A**).

As oncolytic viruses have been reported to induce tumor cell death associated with immunogenic features (17), we further assessed whether KLS-3021 triggers ICD molecular hallmarks. Although exhibiting morphological features commonly associated with apoptotic cell death, this form of cell death is functionally distinct from classical apoptosis in its capacity to release or expose DAMPs such as high-mobility group box 1 (HMGB1) and calreticulin (CRT), which promote immune recognition of dying tumor cells. In vehicle- and docetaxel-treated tumors, HMGB1 expression was weak, whereas KLS-3021-treated tumors exhibited strong HMGB1 expression (**Figure 5B**). Similarly, CRT expression was markedly upregulated in KLS-3021-treated tumors and displayed prominent membrane localization, indicating that surface exposure facilitated immunogenic signaling and phagocytic clearance (**Figure 5B**).

Cumulatively, these data suggest that KLS-3021 induces tumor cell death accompanied by hallmark features of ICD, including increased TUNEL positivity, suppressed proliferation, and upregulation of DAMP-associated markers (HMGB1 and CRT). These features indicate that KLS-3021 not only exerts direct oncolytic effects but also induces ICD, contributing to the establishment of a durable, proinflammatory, and anti-TME in the locally invasive cancer model.

### KLS-3021 spreads to regional lymph nodes and eliminates metastatic tumor cells in a locally invasive orthotopic prostate cancer model

**Figure 6:**
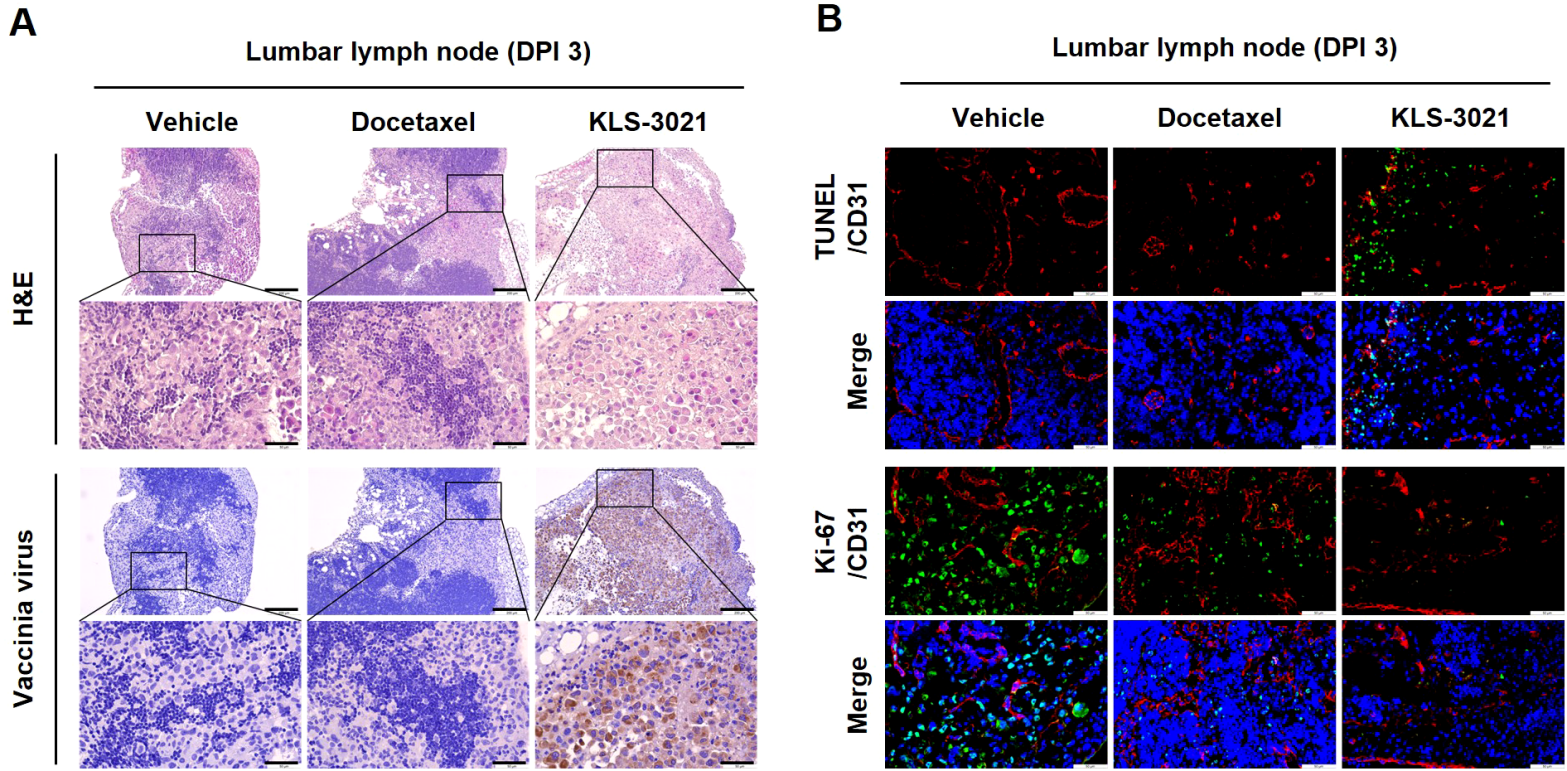
KLS-3021 spreads to regional lymph nodes and eliminates metastatic tumor cells in a locally invasive orthotopic prostate cancer model. **(A)** H&E and vaccinia virus immunohistochemistry staining of lumbar lymph node sections collected on day 3 post-treatment. **(B)** TUNEL/CD31 and Ki-67/CD31 immunofluorescence staining of lumbar lymph nodes collected on day 3 post-treatment. Scale bars, 200 μm. Enlarged scale bars, 50 μm. Representative images from two mice per group are shown.

As an extension of these findings, we examined whether KLS-3021 exerts therapeutic effects beyond the primary prostate tumor in the locally invasive orthotopic prostate cancer model. Histological examination of the lumbar lymph nodes on day 3 post-treatment showed extensive metastatic tumor infiltration in the vehicle- and docetaxel-treated mice. Conversely, lymph nodes from KLS-3021-treated mice exhibited a markedly reduced tumor burden and partial nodal architecture restoration (**Figure 6A**). Immunohistochemical staining for vaccinia virus confirmed viral dissemination within the lymph nodes in KLS-3021-treated mice, indicating that the virus effectively traffics from the primary tumor to regional metastatic sites (**Figure 6A**). Consistent with the tumor-regressive pattern observed in the prostate, TUNEL staining revealed a substantial increase in tumor cell death in the lymph nodes of KLS-3021-treated mice, whereas Ki-67 immunofluorescence showed a pronounced reduction in proliferating tumor cells compared with the control groups. Conversely, CD31 staining revealed no significant differences in vascular structures among the groups (**Figure 6B**).

Together, these findings demonstrate that KLS-3021 effectively reaches the regional lymph nodes and induces tumor cell death at metastatic sites. This capacity for viral dissemination and direct oncolytic activity underscores the potential of KLS-3021 to control not only primary prostate tumors but also regional metastatic disease within the lymphatic system.

## Discussion

Prostate cancer remains a major clinical concern, particularly in aging populations, where disease incidence, prevalence, and complexity are increasing. For patients with low-risk disease, close surveillance is commonly employed; however, the unpredictable nature of disease progression and fluctuating levels of prostate-specific antigen, a widely used biomarker for disease monitoring, can constrain the window for curative intervention. This uncertainty not only complicates clinical decision-making but also contributes to patient anxiety, especially among those seeking early minimally invasive treatment options. In contrast, patients with locally advanced or lymph node-positive disease often face limited therapeutic choices, particularly when comorbidities or advanced age preclude surgery or systemic therapy. These challenges are further compounded by the immunologically “cold” TME characteristic of prostate cancer, marked by low immune cell infiltration and immunosuppressive signaling, which impairs the ICB efficacy. Collectively, these clinical and biological barriers underscore the urgent need for localized, proactive therapeutic strategies capable of circumventing immune resistance and expanding treatment accessibility across disease stages (18, 19).

In this study, we demonstrated that KLS-3021, a novel oncolytic vaccinia virus, exerts potent anti-tumor activity in clinically relevant orthotopic prostate cancer models using PC-3 cells. Notably, a single localized administration of KLS-3021 led to rapid and sustained tumor regression in both prostate-confined (bioluminescence signal-positive, non-palpable tumors) and locally invasive (macroscopically visible tumors) disease settings. The therapeutic effects of KLS-3021, confirmed *via* longitudinal *in vivo* imaging and histopathological analyses, significantly exceeded the efficacy of systemic chemotherapy (docetaxel). Importantly, KLS-3021 not only controlled primary tumor growth but also suppressed lymphatic dissemination, highlighting its potential to address both local and metastatic diseases. While the present work focused on a PC-3–based orthotopic model selected for its relevance to aggressive, androgen-independent disease, KLS-3021 has shown consistent anti-tumor activity across multiple solid tumor models in previous studies, supporting the broader applicability of this therapeutic platform.

The ability of KLS-3021 to achieve local and regional tumor control through a single localized injection represents a key distinction from many previously reported oncolytic viro-immunotherapy approaches in prostate cancer models (20). Prior studies using oncolytic viruses, including vesicular stomatitis virus–based platforms, have often relied on repeated dosing schedules or combination regimens with systemic therapies to achieve meaningful anti-tumor responses, particularly in metastatic or subcutaneous models (21–25). In contrast, KLS-3021 demonstrated robust efficacy following a single intratumoral or intraprostatic administration, suggesting that its integrated design may overcome intrinsic barriers within the prostate tumor microenvironment that otherwise necessitate repeated or combinatorial treatment strategies.

Mechanistically, KLS-3021 exerts its anti-tumor effects through a multifaceted strategy. KLS-3021 is an engineered oncolytic vaccinia virus encoding PH-20, IL-12, and sPD1-Fc, and the effects of each transgene in KLS-3021 were evaluated in the prior published study (11). The objective of the present study was not to dissect the functional mechanism of each transgene, but rather to evaluate the therapeutic performance of KLS-3021, a clinical candidate, in anatomically and pathologically relevant orthotopic prostate cancer models. The initial action of KLS-3021 involves tumor-selective direct oncolysis and then PH-20-mediated hyaluronan degradation, which substantially remodels the dense ECM, a physical barrier that impedes viral dissemination and immune cell infiltration (26). This ECM remodeling facilitated widespread intratumoral viral distribution and enabled KLS-3021 to reach the regional lumbar lymph nodes harboring metastatic tumor cells. Histological analyses confirmed the viral presence and anti-tumor activity at both the primary and nodal sites, indicating that KLS-3021 can extend its therapeutic reach beyond the injection site *via* viral spread. This physical remodeling establishes a permissive microenvironment for both viral propagation and immune cell recruitment.

In parallel, KLS-3021 triggered a potent and orchestrated immunomodulatory response, shifting the tumor immune microenvironment toward a proinflammatory state that is associated with favorable immunological features linked to therapeutic responsiveness. Histological analyses revealed a marked increase in F4/80^+^CD86^+^ macrophages, indicative of M1-like polarization, alongside a reduction in CD206^+^ M2-like macrophages. This repolarization was accompanied by elevated TNF-α expression, reinforcing the establishment of an inflammatory milieu. While the present study was not designed to establish a causal requirement for macrophages in mediating tumor control, these findings showed a shift toward a proinflammatory microenvironment that has been associated with improved prognosis and enhanced immunotherapeutic efficacy across multiple cancer types (27). Collectively, these immune alterations can be interpreted as part of a broader treatment-associated remodeling.

This proinflammatory shift was accompanied by tumor cell death associated with ICD induction, as evidenced by increased TUNEL-positive signals and the release of DAMPs, including HMGB1 and CRT (28), which are critical for the activation of type 1 conventional dendritic cells that orchestrate adaptive immune responses through the cross-presentation of tumor antigens to naïve T cells (29). Although these studies were conducted in T-cell-deficient nude mice, limiting the direct assessment of adaptive immunity, the observed cascade—from ECM remodeling to M1 macrophage polarization and ICD—strongly supports the immunostimulatory potential of KLS-3021 in immunocompetent settings.

The translational relevance of our findings is strengthened by the use of two orthotopic prostate cancer models: an invisible, non-palpable, bioluminescence-positive, prostate-confined model, and a locally invasive model with gross tumors and regional lymphatic spread. These models were selected to closely mimic the anatomical and pathological progression of human prostate cancer, enabling a clinically relevant and multifaceted evaluation of KLS-3021 in both early and advanced disease contexts. By capturing key clinical scenarios, including localized diseases suitable for minimally invasive interventions, and regionally advanced diseases with limited treatment options, these models provide a robust preclinical framework for assessing the therapeutic potential of KLS-3021. Notably, they reflect the clinical needs of older or medically ineligible patients, underscoring the promise of KLS-3021 as a minimally invasive or neoadjuvant therapeutic candidate aimed at improving outcomes in this underserved population. From a clinical perspective, KLS-3021 may be particularly relevant for prostate cancer patients who could benefit from localized, minimally invasive therapeutic strategies. These may include patients with prostate-confined disease under active surveillance seeking earlier local intervention; patients with locally invasive tumors for whom definitive local therapies or systemic treatments are not immediately suitable; and patients with more advanced disease in whom tumor volume reduction may help alleviate urinary symptoms while minimizing treatment-related morbidity.

Several limitations of this study should be acknowledged. All *in vivo* experiments were conducted in athymic nude mice, precluding direct evaluation of adaptive T cell–mediated immune responses. Accordingly, immune-related changes within the tumor microenvironment were evaluated with a focus on innate immune activation. In addition, a small number of animals in the KLS-3021–treated group died during the locally invasive model study. Based on post-mortem gross examinations, these events were considered most consistent with anatomical and model-specific factors, such as urinary tract obstruction associated with tumor implantation or local tissue changes, rather than treatment-related systemic toxicity. Although systemic viral dissemination is a theoretical concern in immunodeficient hosts, prior biodistribution and toxicity studies in immunocompetent mice demonstrated rapid viral clearance and no evidence of sustained systemic toxicity. Furthermore, no clinical signs indicative of viral toxemia were observed in the affected animals. Taken together, these observations suggest that the mortality observed in this model is unlikely to reflect intrinsic systemic toxicity of KLS-3021 or to be predictive of clinical risk.

Collectively, our findings provide compelling preclinical evidence that KLS-3021 exerts potent anti-tumor activity in both primary and locally invasive prostate cancers through the synergistic actions of ECM remodeling, innate immune activation, and ICD induction. These results suggest that KLS-3021 is a promising next-generation oncolytic immunotherapy that may serve as a minimally invasive or neoadjuvant therapeutic strategy for prostate cancer, particularly in older or medically ineligible patients who are unsuitable for surgery or systemic therapy. Its integrated multifactorial mechanism of action and localized delivery strategy offer unique advantages in terms of both efficacy and safety, laying the groundwork for future clinical development.

## Data Availability

The raw data supporting the conclusions of this article will be made available by the authors, without undue reservation.
